# Dogs and the city. The role of urban landscape for dog walking habits, and health benefits for people and dogs

**DOI:** 10.1186/1751-0147-57-S1-O20

**Published:** 2015-09-25

**Authors:** Ingrid Sarlöv Herlin

**Affiliations:** 1 Department of Landscape Architecture, Planning and Management, Swedish University of Agricultural Sciences, Uppsala, Sweden

## Introduction

The highest proportion of dogs, in Sweden, is found in urbanized counties. Today's sedentary lifestyle leads to worsened health conditions and higher abundance of diabetes and cardiovascular diseases among humans and dogs. While dog ownership leads to an increase in recreational walking is not clear how the urban landscape can contribute to motivation and perceived access.

## Objectives

This study investigates how cities and the urban landscape can be better planned and designed to support dog walking, and through this promote physical and social wellbeing among people and animals.

## Methods

This interdisciplinary study is based on a literature review of relevant fields, such as different approaches to ‘walkability’ and landscape perception; relations between physical environment and human and animal health; animal human interactions; and planning and management of urban green structure and public spaces.

## Results

Preliminary results show that regular outdoor activities and exercise are positively correlated with people's mental and physical health status, and dog walking has been shown as a viable method for physical activity. The concept of green infrastructure stresses the need for well-connected, easy-accessible, multifunctional green networks. The quality of urban green infrastructure and the experiences they offer are important for enhancing recreational use and physical activity.

## Conclusions

Planning, designing and managing and attractive, diverse and well-connected green infrastructure for everyday activities is an important, but neglected task for green space planners and managers that contribute to health and well-being for animals and people.

